# Trust and cancer screening: Effects of a screening controversy on women’s perceptions of cervical cancer screening

**DOI:** 10.1016/j.pmedr.2021.101684

**Published:** 2021-12-27

**Authors:** B. O'Donovan, Therese Mooney, Ben Rimmer, Patricia Fitzpatrick, Grainne Flannelly, Lorraine Doherty, Noirin Russell, Cara M. Martin, John J. O'Leary, Linda Sharp, Mairead O'Connor

**Affiliations:** a Trinity St. James's Cancer Institute, Ireland; b Department of Histopathology, Trinity College Dublin, Ireland; c Department of Pathology, Coombe Women and Infants University Hospital, Ireland; d National Screening Service, Dublin, Ireland; e Newcastle University Centre for Cancer, Newcastle-upon-Tyne, UK; f University College Dublin, Ireland; g National Maternity Hospital, Dublin, Ireland; h University College Cork, Cork, Ireland

**Keywords:** Screening, Cervical cancer, Trust, Perceptions, Qualitative

## Abstract

There is a paucity of data on trust of service users in cervical screening. A significant controversy in Ireland’s national cervical cancer screening programme emerged in 2018. The Health Service Executive (HSE) confirmed that a clinical audit had revealed that more than 200 women who developed cancer had not been told of earlier misdiagnosed smear tests. During this high profile controversy we conducted qualitative interviews exploring factors that influence cervical screening participation. Women who had been invited for routine screening tests were recruited from the national screening register. Telephone interviews were conducted with 48 women aged 25–65 years; with a range of screening histories – 34 were adequately screened (attended all routine screening tests) and 14 were inadequately screened (attended some/no screening tests). Thematic analysis was conducted and all interviewees spontaneously raised the screening controversy revealing that the crisis had resulted in serious loss of trust, faith and confidence in the screening programme. Publicity surrounding the controversy had some beneficial effects, including increased awareness of the value of screening and beliefs that intense focus on the programme will improve the service long-term. Strategies which incorporate these findings could help rebuild trust in screening.

## 1 Background

Trust of service users in screening is a complex issue. Organised screening programmes have contributed to decreases in cervical cancer incidence and mortality internationally (World Health Organization, 2018). To achieve these benefits, high participation is essential (Yang et al., 2011); to regularly attend, women must trust the service. Breast and colorectal cancer screening research, conducted in different healthcare systems, has found that distrust of healthcare systems and/or professionals can be barriers to participation (Yang et al., 2011, Ward et al., 2015 Sep, Clarke et al., 2016). Data on trust in cervical screening is limited.

In Ireland, in 2018, a controversy emerged in the cervical screening programme, CervicalCheck. The Health Service Executive (HSE) confirmed that smears from 206 women had been changed upon review to a different finding or a warning of increased risk/evidence of developing cancer. Most women had not been told of these findings. A Scoping Inquiry into CervicalCheck, conducted by Dr Gabriel Scally was commissioned in 2018 and a screening history review by Royal College of Obstetricians and Gynaecologists (RCOG) was published in 2019. Both confirmed that CervicalCheck was operating at international standards and although the Scoping Inquiry (Scally review) was critical of programme governance, it found no evidence of laboratory underperformance (Government of Ireland, 2018, RCOG Independent Expert Panel, 2019). On direction from the Minister for Health, free “out of cycle” smear tests were offered by the HSE to any woman who had a prior CervicalCheck smear test. Research has been conducted with women and their partners who were directly affected by the controversy (Lynch et al., 2021, D’Alton et al., 2021). These studies focused on the psychological impact on members and partners of the ‘221 + Patient Support Group’; to date, no research has explored the effect of the controversy on women’s views and attitudes towards cervical screening.

In 2019 we conducted a qualitative study on influences on cervical screening participation (O’Donovan et al., 2021). We interviewed women with a variety of ages and screening histories from the general population. The aim of this paper was to use a unique opportunity provided by circumstance to examine the impact of a high-profile controversy on women’s current views of cervical screening and their future screening intentions.

## 2 Methods

Methods are described in detail elsewhere (O’Donovan et al., 2021). In brief, since 2008, CervicalCheck has offered free cervical screening tests to women in Ireland aged 25–65 years. Women on the CervicalCheck screening register are issued invitation letters to remind them when their next screening test is due. They may book their test at any CervicalCheck registered GP or clinics. A sampling frame was generated from the CervicalCheck screening register. Women diagnosed with cervical cancer, those undergoing colposcopy clinic surveillance for abnormal cytology, and those still awaiting results from a free “out of cycle” test were excluded. A priori, it was decided to use purposive sampling to select potential study participants from the register; sampling strata were age (<50; 50+) and cervical screening history (adequate, that is had attended all routine screening tests they had been invited to since programme inception /inadequate, had attended none or some routine screening tests they had been invited to). This produced four strata – older and younger women who were adequately screened and older and younger women who were inadequately screened. In April 2018, CervicalCheck staff selected women filling each strata at random from the population register (600 women in total); selected women were invited to participate. Data collection, coding and analysis were conducted by researchers independent of CervicalCheck. Semi-structured telephone interviews were conducted August-December 2019. Women who were willing to participate completed and signed a consent form before interview. The topic guide primarily sought to explore decision making around screening participation, but also included questions on awareness and experiences of screening and the controversy. Inductive thematic analysis was conducted (Braun et al., 2012). Saturation (defined as no new issues or themes emerging in the last three interviews) was reached after 48 interviews. The Royal College of Physicians of Ireland provided ethical approval (RCPI RECSAF 74-2).

## 3 Results

Forty-eight women were interviewed (Supplementary Table 1) of whom 34 were adequately screened and 14 were inadequately screened. All women raised the screening controversy without prompting, some at the very start of the interview. All expressed strong opinions. Five themes were identified: (1) perceptions of cervical screening; (2) emotional impact of the controversy; (3) future screening intentions; (4) positive effects of the controversy; and (5) unmet information needs. Results, and illustrative anonymised quotes, are in Supplementary Table 2.

### 3.1 Perceptions of cervical screening

Women spoke about how the controversy had greatly worried them and damaged their trust in cervical screening. They considered that screening had many benefits; notably providing a free service which saved lives. However, many described a significant loss of faith and confidence in the system due to the controversy. Many adequately screened women felt strongly that they had lost the ‘peace of mind’ and reassurance which they had associated with attendance in the past and had greatly valued. Concerns about the accuracy of test results – and specifically that something may have been missed/misread - had eroded their previous trust and led to them questioning the value of screening.

### 3.2 Emotional impact of the controversy

All women (irrespective of their screening history) expressed strong opinions about the controversy and considered it had caused considerable distress, worry and upset for themselves and other women. Using emotive terms like “appalling” and “terrible”, they described how their feelings of frustration, sadness, anger and confusion increased as events unfolded.

Women also discussed their personal experience of the “out of cycle” tests offered by the HSE. They described long waits for test appointments, their concerns about the laboratories where tests were being processed and prolonged delays awaiting results, as a backlog developed. Many reported that these experiences exacerbated their anxiety and frustration. Some highlighted the unnecessary anxiety created by women being offered repeat smears and having to wait for results when ultimately there is “nothing wrong” with them.

### 3.3 Future screening intentions

Despite the controversy most adequately screened women intended to attend cervical screening in the future. However, they considered that lack of confidence in screening resulting from the controversy might discourage other women from attending. Most inadequately screened women described being more aware of screening, but did not report intending to attend more regularly in future. Some said they needed reassurance that the screening service is being “run right”. Many women (both adequately and inadequately screened) expressed concerns about the reliability of cervical screening; they questioned the accuracy of their past screening tests as well as those they might have in the future.

### 3.4 Positive effects of the controversy

Several women described how publicity surrounding the crisis had placed more information about screening in the public domain and how this had increased their belief in the importance and value of screening. Many women (both adequately and inadequately screened) felt that the attention on the programme would have long-term benefits and ultimately improve the service; for example, that there would be closer monitoring of laboratories and increased transparency for service users.

### 3.5 Unmet information needs

Some adequately and inadequately screened women spoke about being unclear about the purpose of screening and displayed limited cervical cancer knowledge. Many revealed they did not understand the screening terminology being widely used in the media at that time (e.g. some spoke about ‘false positives’ or ‘false negatives’ but used these terms incorrectly). Women talked about the need for accessible, reliable information to address confusion over screening and its purpose in general, and clarification of screening language and terminology.

## 4 Discussion

The countries of Europe are adopting a variety of strategies to reach the WHO goal of eliminating cervical cancer as a public health problem; cervical screening is a cornerstone of these strategies (WHO, 2018) and trust among service users is key to the success of screening programmes. As was shown in the UK twenty years ago, high-profile controversies within cervical screening programmes can have adverse effects on women’s trust in screening (Houston et al., 2001). Our findings – which show a striking loss of faith, trust and confidence in screening (past, present and future) following the controversy in Ireland’s cervical screening programme – echo this.

Each woman’s results letter from CervicalCheck states that “no screening test is 100% effective”. However it became clear as the crisis developed that the general public and media in Ireland did not fully understand the differences between screening and diagnostic tests. This general lack of understanding was echoed in our finding that, despite being very aware of the controversy, participants often demonstrated a misunderstanding of the purpose of screening or used screening terminology incorrectly. This suggests there is a need for initiatives to improve people’s understanding of screening and its purpose; to support and enable informed decision-making around participation.

Our findings show how deficiencies in communication and mishandling of information, resulted in significant distrust; this was compounded by the fallout from the Minister of Health’s decision to offer free “out of cycle” smear tests, which led to major backlogs in the laboratories and long delays for women getting results. Other authors have observed that cervical screening can have unintended adverse consequences – such as distress related to cytological follow up of mildly abnormal screening tests **-** for women (Sharp et al., 2014). The current study provides a further illustration of this.

The RCOG review concluded that the Irish Cervical Screening Programme was performing at international standards and had a similar rate of slide discordance on retrospective review to the UK cervical screening programme (RCOG Independent Expert Panel, 2019). However we found women’s loss of trust in the programme was often linked to concerns about test accuracy, including past tests and those they might have in the future. Communication strategies which offer reassurance about the reliability of tests could address these concerns and encourage women to attend their future screening appointments.

The impact of the controversy on screening uptake in Ireland is not yet fully clear but, as these results suggest, it will be essential to restore trust in screening. While women were aware of potential health benefits of screening, many focused on the loss of emotional benefits, particularly, the reassurance of a negative test. This suggests that interventions to rebuild trust might target emotional, as well as health, benefits of screening.

Interestingly, it appears that some positive consequences could follow from publicity surrounding the controversy. There was a greater awareness of the importance of screening among women as well as a belief that the intense focus on the programme would result in an improved service long-term. These positive aspects may provide a foundation upon which to re-establish public confidence and reduce screening barriers.

The main limitation of the study is one that many research studies share - participants generally have an interest in the topic; here they may have been motivated to take part as they had particular views about screening. In addition, the interviews were conducted while the high-profile controversy around CervicalCheck was being extensively reported across media outlets. This may also have impacted on women’s views on screening and our findings. The cross-sectional design means the results represent a single snapshot in time. A key strength is that the design included women with a variety of screening histories; to be eligible women had to be invited for screening by CervicalCheck between the start of the programme (Sept 2008) and March 2018. This ensured the views of inadequately screened women, an under-studied group, were captured.

## 5 Conclusions

Women’s perceptions of, and trust in, cervical screening have been adversely affected by this screening controversy. These findings can be used to inform the development of strategies to address women’s concerns and start to rebuild trust in screening.

## Declaration of Competing Interest

The authors declare that they have no known competing financial interests or personal relationships that could have appeared to influence the work reported in this paper.
