# Supplementary material for: Trust and cancer screening: Effects of a screening controversy on women’s perceptions of cervical cancer screening
Source: Prev Med Rep. 2021 Dec 27;25:101684. doi: 10.1016/j.pmedr.2021.101684 (PMC8800010; doi:10.1016/j.pmedr.2021.101684)
Supplement: Supplementary data 1 [file mmc1.docx]

**Supplementary table 1**

*Table 1: Characteristics of study participants (n=48)*

| **Demographics** | **Number (n)** | **Demographics** | **Number (n)** |
| --- | --- | --- | --- |
| Age at interview (years)  **25-40**  41-50  51-60  **61-65^*^** | 9  11  24  4 | Education  Tertiary level^**^  Diploma/certificate  Leaving certificate**^***^** | 22  15  11 |
| Screening history  Adequately screened^#^  Inadequately screened^##^ | 34  14 | Employment  Employed/self employed  Retired  Unemployed  Other | 38  3  2  5 |
| Relationship status  Married/Cohabiting  Separated  Divorced  Single | 36  5  3  4 | Previous abnormal cytology result(s)^~^  Yes  No | 34  14 |

***Maximum age in this range = 62 years**; **Post secondary education e.g. universities, higher education institutions; ***School leaving qualification; **#attended all routine CervicalCheck screening tests (confirmed by self-report at interview); ## attended some or no CervicalCheck screening tests (confirmed by self-report);** ~self-reported previous abnormal smear within CervicalCheck

**Supplementary table 2**

*Table 2*: *Impact of screening controversy – themes, main issues & illustrative quotes (n=48), with indication of whether the issue arose in adequately screened (AS) or inadequately screened (IS) women or both groups**

| **Themes and main issues** | **Quotes** |
| --- | --- |
| **Perceptions of screening programme**   - loss of trust, faith and confidence in programme among women (AS & IS) | “Definitely my views [on screening] will have changed completely. I definitely wouldn’t be as trusting at all now, or believe as much that it is doing what it should be doing”  (DM550487, AS, 31 yrs)  “I think people have no faith in that system, including myself. At the back of your mind is, is that definitely my result? Can I be sure that that is my result?”  (DS30036, AS, 50 yrs)  “the overriding thing at the minute when we hear CervicalCheck and smears is that we don’t trust the system that’s in place for it, it just seems to be one disaster after the other and no accountability for it. Hopefully that’s something that changes but I don’t know, I don’t have a whole lot of faith” (DM550498, IS, 32 yrs) |
| **Emotional impact of the screening controversy**   - feelings of anger, confusion, frustration (AS & IS) - issues related to repeated smear tests offered by the HSE:   delays in getting appointments for rechecks and long waits for results (AS)  concerns about laboratories (AS)  unnecessary anxiety created by repeat smears (IS) | “No one seems to be held accountable for what happens. People have died. It is awful”  (DS300026, AS, 43 yrs)  “It was shocking what happened”  (DS300024, AS, 61 yrs)  “In my last one [smear test] it took five months before I got my results back, which seems like very long”  (DM550019, AS, 60 yrs)  “In terms of negatives, for some people it could cause a lot of anxiety because they might need repeat smears when there's nothing wrong”  (DM550498, IS, 32 yrs)  “I regret that I didn’t ask the nurse at the time, ‘Where is it [smear test] going?’ I don’t know where it’s going to be tested, and I wish I had asked that”  (DM550490, AS, 54 yrs) |
| **Effects on future screening behaviours**   - intention to keep attending in the future (AS) - concerns that lack of confidence in screening among women could result in lower attendence (AS) - need reassurance to address doubts about service (IS) - concerns about the reliability of cervical screening (AS & IS) | “I will definitely keep doing them [smear tests], and I understand that there’s problems but I must have confidence that they’re going to fix them”  (DM550492, AS, 34 yrs)  “It [the controversy] is such a shame, because when it is done properly, it is an amazing service. It saves lots and lots of lives but whatever happened here, it has just been appalling. I think there is a total lack of confidence. That will definitely deter women from going”  (DS300036, AS, 50 yrs)  “To be assured that when you do spend the half hour or whatever going for it [smear test], that you can walk away like you used to and not think about it again”  (DM550499, IS, 56 yrs)  “Peoples’ minds need to be put at ease, that everything is okay [with] the service”  (DS300017, IS, 52 yrs)  “I would still go and do it but every result I get, I’d take with a pinch of salt”  (DM550487, AS, 31 yrs) |
| **Positive effects of controversy**   - greater awareness of screening as a result of publicity (AS & IS) - potential for an improved service long-term (e.g. more monitoring of laboratories) (AS & IS) | “I am starting to realise the importance of it [screening]…it has been highlighted all over the news and seeing the situation, everything that has happened with those women… I suppose it [going to appointments] is just as much for them.”  (DS300028, AS, 39 yrs)  “I suppose I would be more aware of going for tests because of all the publicity”  (DM550496, IS, 57 yrs)  “I feel like the government will do something about it [laboratories] because it’s in the media so much”  (DM550492, AS, 34 yrs) |
| **Unmet information needs**   - confusion as to the purpose of screening (AS & IS) - limited knowledge of cervical cancer risk (AS & IS) - lack of understanding of screening terminology (AS & IS) | “And I for one didn’t know that it could not mean that you are healthy if you get a normal report from CervicalCheck…I didn’t actually know. I thought once you get the test done, you're fine.”  (DS300033, AS, 43 yrs)  “I would like a bit more information on that [abnormal result]..A bit more in depth information about that [abnormal result] because in some ways I thought that was detected all those years ago and now I am getting clear results back, then this happened.”  (DS30028, AS, 39 yrs)  “I don’t know what reasons cause it [cervical cancer]”  (DM550494, IS, 50 yrs)  “I went around in circles on the internet to find what I wanted [definition of screening terms]”  (DS300013, IS, 59 yrs) |

**AS, adequately screened, attended all routine CervicalCheck screening tests (confirmed by self-report at interview); IS, inadequately screened, attended some or no CervicalCheck screening tests (confirmed by self-report)**
