# Supplementary material for: Trust and cancer screening: Effects of a screening controversy on women’s perceptions of cervical cancer screening
Source: Prev Med Rep. 2021 Dec 27;25:101684. doi: 10.1016/j.pmedr.2021.101684 (PMC8800010; doi:10.1016/j.pmedr.2021.101684)
Supplement: Supplementary data 2 [file mmc2.pdf]

Topic Guide

• **Introduction**

"Hello…it's XX from xx, we spoke last week and arranged to talk about your views on smear tests and cervical screening..is it still a good time for you?"

Verbal consent

"Just to remind you…"

Start recording

"I'll start the tape now…"

Assure of confidentiality

"Please be assured that everything you say.."

Summarize purpose and structure again

"Before we start I'ld just like to tell you a bit about the study. We are exploring womens' views of smear tests and cervical screening and what influences their decisions to attend for screening tests. We want to hear the views of a variety of women – so women who attend regularily, those who attend sometimes and those who never attend. We also want to find out what women think would improve screening/smear tests. "

So how does that sound? Do you have any questions before we begin?

• **Background (demographic information)**

"So to start off can you tell me a bit about yourself..(raised questioning tone & pause)?"

• Cervical screening history

“Have you ever received an invitation to take part in CervicalCheck?”

If not

“Has your doctor/nurse at your GPs surgery ever mentioned having smear tests?”

“What were your first thoughts when you got your first invitation letter from CervicalCheck?”

“Did these thoughts/your thinking change when you got your second invitation?”

”Did you make arrangements to have a smear test?...Was it long after getting your invitation?”

“Why did you decide to have your screening test?”

“What did you consider when you were making your decision?”

“Did you talk to anyone about it?”

If not

“Why did you decide not to have your CervicalCheck screening test?”

“What did you consider when you were making your decision?”

“Did you talk to anyone about it?”

“Did you know about/were you aware of cervical screening before you got the CervicalCheck invitations?”

“Have you participated in other screening programmes e.g. BreastCheck, Diabetic RetinsScreen?...Can you tell me about that?”

- **Undergoing the screening test**

"So just to return to your most recent screening test..can you tell me about it?"

"What do you think would make undergoing the screening test easier?"

"Was there anything particularily difficult about undergoing the test?"

[Physical/Psychological elements]

"What might make you more likely to participate in screening?.....There is a screening tool that you could use yourself at home called HPV self-sampling. Have you heard of it?"

If not

"So I know you decided not to have your CervicalCheck smear test but I'ld like to ask you some general questions about screening. What do you think would make undergoing the screening test easier?"

"What might make you more likely to participate in screening?...There is a screening tool that you could use yourself at home called HPV self-sampling. Have you heard of it?"

- Information seeking and social support

"If you were looking for trustworthy/reliable information on cervical screening what would you do?...Would you use any other sources of information e.g. friends/medical book/Internet?"

- HPV

"Have you heard about HPV?"

"What can you tell me about it?"

"Have you heard of HPV testing/a HPV test?"

CervicalCheck may soon be changing their screening test from the current test, which looks for abnormal cells in the cervix to a test which looks for the presence or absence of HPV infection. Physically it will feel the same as having smear tests. The changes, if implemented, will mean that all women (aged 25-60 yrs) will attend for screening every 5 years. This will be different from current screening protocols where women aged 24-44 yrs attend for screening every 3 years.

"Do you have any thoughts on the proposed changes?"

Do you think you will be more or less likely to participate in cervical screening in the future if these changes are made?"

• Future improvements to cervical screening

"Looking back what would you have liked to know before you went for your smear test?"

"What would you want a family member/friend who was invited for a smear test to know?"

What would you want a family member/friend who was going for a smear test to know beforehand?"

"What would you want her to know afterwards?...How do you think she should get this information?"

"Any suggestions on how the screening test experience could be improved for other women?"

If not

"So I know you decided not to have your CervicalCheck smear test but I'ld like to ask you some general questions about improving screening…..Looking back to when you were invited

for a smear test (pause) was there anything/any information you would have liked to know?"

"What would you want a family member/friend who was invited for a smear test to know?"

What would you want a family member/friend who was going for a smear test to know beforehand?"

"What would you want her to know afterwards?...How do you think she should get this information?"

"Any suggestions on how the screening test experience could be improved for other women?"

• Close

"Anything else you would like to tell me about cervical screening?"

Thank interviewee. Reassure again about confidentiality and repeat information provided at the beginning. Tell participant what to do if they have any questions/a list of further information can be emailed or posted to her if she requires."
